# Association of health insurance status with presentation, treatment and outcomes in soft tissue sarcoma

**DOI:** 10.1002/cam4.2441

**Published:** 2019-09-04

**Authors:** Varsha Jain, Sriram Venigalla, Ronnie A. Sebro, Giorgos C. Karakousis, Robert J. Wilson, Kristy L. Weber, Jacob E. Shabason

**Affiliations:** ^1^ Department of Radiation Oncology Perelman School of Medicine at the University of Pennsylvania Philadelphia PA USA; ^2^ Department of Radiology Perelman School of Medicine at the University of Pennsylvania Philadelphia PA USA; ^3^ Department of Surgery Perelman School of Medicine at the University of Pennsylvania Philadelphia PA USA; ^4^ Department of Orthopedic Surgery Perelman School of Medicine at the University of Pennsylvania Philadelphia PA USA

**Keywords:** soft tissue sarcoma, radiation therapy, insurance, survival, medicare, medicaid

## Abstract

**Background:**

Numerous studies across a variety of malignancies have demonstrated that health insurance status is associated with differences in clinical presentation, type of treatments received, and survival. The effect of insurance status on the management of soft tissue sarcoma is unknown. We assessed the association of insurance on (a) stage at diagnosis, (b) receipt of neoadjuvant/adjuvant radiation therapy, and (c) overall survival (OS) in patients with soft tissue sarcoma.

**Methods:**

The study cohort was identified from the National Cancer Database (NCDB) and consisted of patients with stage I‐IV soft tissue sarcoma of various histologies diagnosed from 2004 to 2015. The patients were stratified by age (<65 and ≥65 years) and by insurance status (commercial, Medicare, Medicaid and uninsured). Using multivariable logistic regression analysis, we evaluated the association between insurance status and (a) stage at diagnosis (Stage I‐III vs IV), and (b) receipt of neoadjuvant/adjuvant radiation therapy in patients with locally advanced disease. The association of insurance status on OS was assessed using Kaplan‐Meier and multivariable Cox proportional hazards analyses. A propensity score matched survival analysis was performed to account for measured confounders.

**Results:**

49 754 patients were identified of whom 23 677 (48%) had commercial insurance, 20 867 (42%) had Medicare, 3229 (6%) had Medicaid, and 1981 (4%) were uninsured. In patients <65 years, those with Medicaid (OR = 1.74, 95% CI: 1.57‐1.93, *P* < .001) and the uninsured (OR = 1.71, 95% CI: 1.51‐1.94, *P* < .001) were more likely to present with stage IV vs Stage I‐III disease. Furthermore, among patients with locally advanced disease treated with limb sparing surgery, those with Medicaid (OR = 0.87, 95% CI: 0.77‐ 0.98, *P* = .021) and the uninsured (OR = 0.73, 95% CI: 0.63‐0.85, *P* < .001) were less likely to receive neoadjuvant or adjuvant radiotherapy as compared to those with commercial insurance. Lastly, having Medicaid (HR = 1.26, 95% CI: 1.17‐1.34, *P* < .001) and no insurance (HR = 1.30, 95% CI: 1.20‐1.41, *P* < .001) was associated with worse OS compared to having commercial insurance, a finding which remained significant after propensity score matching. In contrast, in patients ≥65 years, there were no statistically significant differences between those with Medicare and commercial insurance with regards to disease presentation, receipt of radiotherapy, or survival.

**Conclusions:**

In a large modern cohort identified from the NCDB, commercial insurance status in patients <65 years was associated early diagnosis, receipt of neoadjuvant/adjuvant radiation therapy, and overall survival for patients with soft tissue sarcoma. Further efforts are warranted to understand disparities in care based on health insurance in the United States.

## Graphical abstract

Using a national cancer registry, we demonstrate that insurance status is associated with differences in stage of diagnosis, receipt of neoadjuvant or adjuvant radiation therapy, and overall survival for patients with soft tissue sarcoma in the United States. These insurance related disparities are most prominent in Medicaid and uninsured patients <65 years of age.

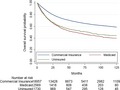

## 1 INTRODUCTION

Soft tissue sarcomas (STS) are rare tumors representing less than 1 percent of all newly diagnosed cancers in the United States.^1^ As STS's are uncommon and often complex, a multi‐disciplinary treatment approach and adherence to evidence based recommendations is particularly important. A National Cancer Database (NCDB) study, analyzing data from 15 957 patients with STS showed that guideline adherent treatment was associated with improved survival outcomes.^2^

For many patients, health insurance coverage affects access to specialized cancer care with important clinical consequences. Studies from several disease sites have shown that health insurance coverage influences receipt of guideline recommended care, timely treatment, as well as participation in clinical trials.^3^ For instance, a recent study evaluating the impact of insurance coverage on outcomes of patients with breast sarcoma showed that Medicaid and uninsured patients were more likely to present with advanced disease and have worse outcomes as compared to privately insured patients.^4^ Other studies in adult patients with STS have similarly demonstrated that noncommercial insurance is associated with a longer time to treatment initiation^5^ and a lower likelihood of receiving guideline concordant care^2^ as compared to privately insured patients.

The aim of this study was to utilize the NCDB to evaluate the association between insurance status and (a) stage of disease at diagnosis, (b) receipt of neoadjuvant or adjuvant radiation and (c) overall survival (OS) among patients diagnosed with STS.

## 2 METHODS

### 2.1 Data source

The study population was identified from the NCDB, a national cancer registry jointly sponsored by the American College of Surgeons and the American Cancer Society that draws upon hospital registry data from more than 1500 Commission on Cancer (CoC)‐accredited facilities in the United States.^6^ The dataset captures more than 70% of incident cancers and comprises more than 34 million unique cancer cases.

### 2.2 Study population

**Figure 1:**
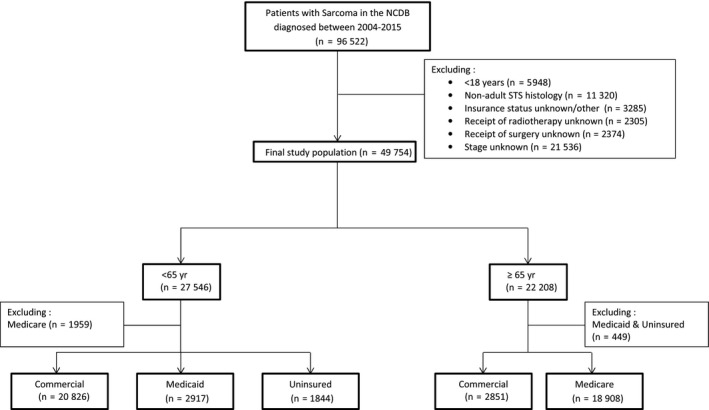
Consolidated Standards of Reporting Trials (CONSORT) diagram of the patient cohort; NCDB, National Cancer Database; STS, Soft Tissue Sarcoma

Inclusion criteria consisted of patients ≥18 years of age with American Joint Committee on Cancer 8th edition stage I‐IV STS, who were diagnosed between 2004 and 2015**.** The study population consisted of STS histologies standardly treated with surgery and radiation, such as undifferentiated or unclassified sarcoma, undifferentiated pleomorphic sarcoma, liposarcoma, leiomyosarcoma, fibrosarcoma, synovial sarcoma, angiosarcoma and malignant peripheral nerve sheath tumors. Retroperitoneal sarcomas as well as histologies primarily encountered in pediatric populations such as rhabdomyosarcoma, or those treated with a different paradigm were excluded. Patients with STS arising in the head and neck, extremities, thorax, abdomen and pelvis were included. Patients were excluded if they had unknown or missing data regarding clinical stage, receipt of surgery, radiation, or insurance status (Figure 1). Of note, the excluded patients with unknown information regarding receipt of radiation, surgery or stage were equally distributed/balanced between the different insurance groups.

### 2.3 Patient cohorts and variables

Patients were stratified by (a) age (< or ≥65 years) and (b) insurance status. A threshold of 65 years was used as it represents the age of eligibility for Medicare insurance. In the cohort of patients <65 years of age, only patients with commercial insurance, Medicaid, and the uninsured were included; those with Medicare were excluded as qualification for Medicare in this age group requires a severe disability or additional comorbidities that we were unable to account for in this analysis. In the ≥65 years cohort, only patients with commercial insurance or Medicare were included as patients with Medicaid and the uninsured comprised a very small proportion of the population (<5%).

The primary independent variable of interest was insurance status. Dependent variables evaluated were (a) stage at diagnosis, (b) receipt of neoadjuvant or adjuvant radiation therapy, and (c) OS. Other covariates examined included age, gender, race/ethnicity, population density of patient residence (metropolitan, urban or rural), type of treatment facility (academic vs. nonacademic), location of treatment facility, patient distance to treatment facility, facility volume (categorized by case volume into four groups; Group 1: 0th‐49th percentile, Group 2: 50th‐89th percentile, Group 3: 90th‐98th percentile, and Group 4: ≥99th percentile), education level (defined as % population in the patient's ZIP code without a high school degree), income (median income in the patient's ZIP code), Charlson/Deyo comorbidity score, year of diagnosis, primary site of tumor, tumor size, tumor histology, type of surgical resection, receipt of radiation therapy (covariate in survival analysis) and receipt of chemotherapy.

### 2.4 Endpoints

In both age cohorts (< and ≥65 years), we evaluated the association between insurance status and three primary endpoints, namely (a) stage at diagnosis (Stage I‐III vs Stage IV), (b) receipt of neoadjuvant or adjuvant radiation therapy for locally advanced STS patients (Stage II/III and non‐metastatic node positive Stage IV) who underwent limb sparing surgery, and (c) OS.

### 2.5 Statistical analysis

Baseline characteristics of patients were stratified by insurance status and compared using Pearson's chi‐squared tests. Covariates achieving a threshold significance of *P* < .1 on univariate analysis were included in the multivariable logistic regression model.

We performed three main analyses. The first analysis included all the patients and utilized a multivariable logistic regression model to assess the association between insurance status and stage at diagnosis (Stage I‐III vs Stage IV).

The second analysis was limited to patients with locally advanced disease (Stage II/III and non‐metastatic node positive Stage IV) who underwent limb sparing surgery. Another multivariable logistic regression model was used to evaluate the association between insurance status and receipt of neoadjuvant or adjuvant radiation therapy.

The third analysis assessed the independent effect of insurance status on hazards of death using Cox proportional hazards analyses. To more robustly account for baseline difference between cohorts, a secondary survival analysis was performed using propensity score analysis. For patients <65 years, a matched cohort of 5138 patients (2569 patients with Medicaid matched with 2569 patients with commercial insurance) was identified using 1‐to‐1 nearest neighbor propensity score matching without replacement.^7^ Propensity scores were derived using multivariable logistic regression methods (matched for all variables in Table S3) and denoted the probability of not having commercial insurance. This was repeated for patients with no insurance (n = 1730). In a similar manner, 2562 patients with commercial insurance were matched to 2562 patients with Medicare in the ≥65 years cohort. Absolute standardized differences of <0.1 between baseline covariates following matching was accepted as a measure of adequate balance.^8^ A Cox survival analysis was then repeated on the matched cohort to estimate the hazard of death associated with insurance status.

For all analyses, a two‐tailed *P* < .05 was considered statistically significant. All analyses were performed using Stata SE, version 15.0 (StataCorp, College Station, TX).

## 3 RESULTS

### 3.1 Baseline clinical characteristics

49 754 patients met study inclusion criteria. Of these, 23 677 (48%) had commercial insurance, 20 867 (42%) had Medicare, 3229 (6%) had Medicaid and 1981 (4%) had no insurance. The median age of the study population was 62 years (interquartile range: 49‐74 years); 27 546 (55%) were <65 years and 22 208 (45%) were ≥65 years. The majority of patients had extremity tumors (53%), localized disease (Stage I: 20%, II: 21%, III: 41%, IV: 18%), underwent limb sparing surgery (78%), did not receive radiotherapy (none: 61%, neoadjuvant: 10%, adjuvant: 29%) nor chemotherapy (75%). The full list of demographic and clinical factors is presented in Table 1.

**Table 1:** Baseline characteristics of the patient cohort

	Commercial (%)	Medicare (%)	Medicaid (%)	Uninsured (%)	Total (%)	*P* (*χ* ^2^)
Total n	23 677 (48)	20 867 (42)	3229 (6)	1981 (4)	49 754 (100)	
Age						<.001
<65 years	20 826 (88)	1959 (9)	2917(90)	1844 (93)	27 546 (55)	
≥65 years	2851 (12)	18 908 (91)	312 (10)	137 (7)	22 208 (45)	
Sex						<.001
Male	12 971 (55)	11 311 (54)	1592(49)	1148(58)	27 022 (54)	
Female	10 706 (45)	9556 (46)	1637 (51)	833 (42)	22 732 (46)	
Race						<.001
NonHispanic White	18 688 (79)	17 558 (84)	1613 (50)	988 (50)	38 847 (78)	
NonHispanic Black	2369 (10)	1711 (8)	790 (24)	402 (20)	5272 (11)	
Hispanic	1346 (6)	815 (4)	574 (18)	490 (25)	3225 (6)	
Other	1274 (5)	783 (4)	252 (8)	101 (5)	2410 (5)	
Patient residence						<.001
Metropolitan	19 803 (84)	16 695 (80)	2642 (82)	1616 (82)	40 756 (82)	
Urban	2748 (12)	3056 (15)	457 (14)	283 (14)	6544 (13)	
Rural	329 (1)	378 (2)	43 (1)	30 (2)	780 (2)	
Unknown	797 (3)	738 (4)	87 (3)	52 (3)	1674 (3)	
Facility location						<.001
East	4308 (18)	4338 (21)	492 (15)	136 (7)	9274 (19)	
South	6370 (27)	7412 (36)	622 (19)	733 (37)	15 137 (30)	
Central	4938 (21)	5583 (27)	484 (15)	284 (14)	11 289 (23)	
West	3618 (15)	3340 (16)	499 (15)	208 (10)	7665 (15)	
Unknown	4443 (19)	194 (1)	1132 (35)	620 (31)	6389 (13)	
Facility type						<.001
Nonacademic	8941 (38)	11 142 (53)	880 (27)	604 (30)	21 567 (43)	
Academic	10 293 (43)	9531 (46)	1217 (38)	757 (38)	21 798 (44)	
Unknown	4443 (19)	194 (1)	1132 (35)	620 (31)	6389 (13)	
Facility volume						<.001
4 (highest)	18 188 (77)	14 402 (69)	2328 (72)	1408 (71)	36 326 (73)	
3	3348 (14)	3674 (18)	535 (17)	362 (18)	7919 (16)	
2	1544 (7)	1930 (9)	247 (8)	149 (8)	3870 (8)	
1 (lowest)	597 (3)	861 (4)	119 (4)	62 (3)	1639 (3)	
Distance to treatment, miles						.050
<40	17 737 (75)	15 712 (75)	2451(76)	1527 (77)	37 427 (75)	
≥40	5666 (24)	4891 (23)	751 (23)	425 (21)	11 733 (24)	
Unknown	274 (1)	264 (1)	27 (1)	29 (1)	594 (1)	
Zip code education level						<.001
≥21%	3130 (13)	3004 (14)	1018 (32)	629 (32)	7781 (16)	
13%‐20.9%	5248 (22)	5185 (25)	985 (31)	563 (28)	11 981 (24)	
7%‐12.9%	8046 (34)	7055 (34)	845 (26)	477 (24)	16 423 (33)	
<7%	6982 (29)	5353 (26)	354 (11)	283 (14)	12 972 (26)	
Unknown	271 (1)	270 (1)	27 (1)	29 (1)	597 (1)	
Zip code median income						<0.001
<38 000	3071 (13)	3374 (16)	957 (30)	562 (28)	7964 (16)	
38 000‐47 999	4718 (20)	5014 (24)	864 (27)	540 (27)	11 136 (22)	
48 000‐62 999	6249 (26)	5594 (27)	783 (24)	450 (23)	13 076 (26)	
>63 000	9347 (39)	6608 (32)	597 (18)	399 (20)	16 951 (34)	
Unknown	292 (1)	277 (1)	28 (1)	30 (2)	627 (1)	
Comorbidity score						<0.001
0	20 490 (87)	15 135 (73)	2634 (82)	1707 (86)	39 966 (80)	
1	2678 (11)	4296 (21)	459 (14)	222 (11)	7655 (15)	
2	392 (2)	1057 (5)	105 (3)	40 (2)	1594 (3)	
3	117 (<1)	379 (2)	31 (1)	12 (1)	539 (1)	
Primary site						<0.001
Head & Neck	1109 (5)	1433 (7)	165 (5)	99 (5)	2806 (6)	
Upper extremity	3054 (13)	2671 (13)	434 (13)	292 (15)	6451 (13)	
Lower extremity	9811 (41)	8109 (39)	1233 (38)	765 (39)	19 918 (40)	
Thorax	2253 (10)	2063 (10)	313 (10)	168 (8)	4797 (10)	
Abdomen/Pelvis	6471 (27)	5577 (27)	908 (28)	546 (28)	13 502 (27)	
Other/NOS	979 (4)	1014 (5)	176 (5)	111 (6)	2280 (5)	
Histology						<0.001
Unclassified	6326 (27)	6686 (32)	1062 (33)	658 (33)	14 732 (30)	
UPS	1684 (7)	2379 (11)	185 (6)	111 (6)	4359 (9)	
Fibrosarcoma/Myxofibrosarcoma	2856 (12)	2249 (11)	312 (10)	233 (12)	5650 (11)	
Liposarcoma	5951 (25)	4240 (20)	548 (17)	379 (19)	11 118 (22)	
Leiomyosarcoma	3655 (15)	3433 (16)	451 (14)	277 (14)	7816 (16)	
Synovial Sarcoma	1381 (6)	277 (1)	279 (9)	154 (8)	2091 (4)	
Angiosarcoma	787 (3)	1172 (6)	116 (4)	51 (3)	2126 (4)	
MPNST	1037 (4)	431 (2)	276 (9)	118 (6)	1862 (4)	
Grade						<0.001
1	5756 (24)	3595 (17)	505 (16)	363 (18)	10 219 (21)	
2	3876 (16)	2765 (13)	480 (15)	327 (17)	7448 (15)	
3	12 510 (53)	12 966 (62)	1843 (57)	1055 (53)	28 374 (57)	
Unknown	1535 (6)	1541 (7)	401 (12)	236 (12)	3713 (7)	
Tumor size (cm)						<0.001
<5	7316 (31)	5957 (29)	690 (21)	439 (22)	14 402 (29)	
5.1‐10	7238 (31)	6527 (31)	988 (31)	559 (28)	15 312 (31)	
10.1‐15	3894 (16)	3419 (16)	646 (20)	379 (19)	8338 (17)	
>15	3860 (16)	3631 (17)	634 (20)	421 (21)	8546 (17)	
Unknown	1369 (6)	1333 (6)	271 (8)	183 (9)	3156 (6)	
Clinical node status						<0.001
Negative	22 633 (96)	19 812 (95)	2923 (91)	1811 (91)	47 179 (95)	
Positive	1010 (4)	1016 (5)	292 (9)	161 (8)	2479 (5)	
Unknown	34 (<1)	39 (<1)	14 (<1)	9 (<1)	96 (<1)	
Clinical M stage						<0.001
Negative	20 429 (86)	17 586 (84)	2436 (75)	1518 (77)	41 969 (84)	
Positive	3224 (14)	3262 (16)	785 (24)	460 (23)	7731 (16)	
Unknown	24 (<1)	19 (<1)	8 (<1)	3 (<1)	54 (<1)	
Clinical stage						<0.001
I	5670 (24)	3484 (17)	482 (15)	353 (18)	9989 (20)	
II	5025 (21)	4590 (22)	466 (14)	306 (15)	10 387 (21)	
III	9274 (39)	9000 (43)	1370 (42)	787 (40)	20 431 (41)	
IV	3708 (16)	3793 (18)	911 (28)	535 (27)	8947 (18)	
Surgery						<0.001
None	3383 (14)	4322 (21)	791 (24)	523 (26)	9019 (18)	
Resection or LSS	19 416 (82)	15 748 (75)	2231 (69)	1336 (67)	38 731 (78)	
Amputation of Limb	878 (4)	797 (4)	207 (6)	122 (6)	2004 (4)	
Radiation therapy						<0.001
None	13 823 (58)	13 137 (63)	2088 (65)	1340 (68)	30 388 (61)	
Pre‐Op RT	2728 (12)	1927 (9)	292 (9)	179 (9)	5126 (10)	
Post‐Op RT	7126 (30)	5803 (28)	849 (26)	462 (23)	14 240 (29)	
Chemotherapy						<0.001
No	16 597 (70)	17 458(84)	2028 (63)	1365 (69)	37 448 (75)	
Yes	6403 (27)	2876 (14)	1101 (34)	563 (28)	10 943 (22)	
Unknown	677 (3)	533 (3)	100 (3)	53 (3)	1363 (3)	
Year of Diagnosis						<0.001
2004‐2007	7334 (31)	6017 (29)	760 (24)	544 (27)	14 655 (29)	
2008‐2011	8014 (34)	6836 (33)	1137 (35)	747 (38)	16 734 (34)	
2012‐2015	8329 (35)	8014 (38)	1332 (41)	690 (35)	18 365 (37)	

Abbreviations: LSS, Limb sparing surgery; UPS, Undifferentiated pleomorphic sarcoma; MPSNT, Malignant peripheral nerve sheath tumor; NOS, Not Otherwise Specified.

### 3.2 Association between insurance status and stage at presentation

In patients <65 years, Medicaid (OR: 1.74, 95% CI: 1.57‐1.93, *P* < .001) and no insurance (OR: 1.71, 95% CI: 1.51‐1.94, *P* < .001) were associated with a higher likelihood of presenting with Stage IV disease as compared to commercial insurance. In contrast, in patients ≥65 years, there was no statistically significant difference between Medicare vs commercial insurance (OR: 0.96, 95% CI: 0.86‐1.07, *P* = .48) (Table 2, Table S1).

**Table 2:** Adjusted odds of Stage IV disease at presentation and receipt of neoadjuvant/adjuvant radiotherapy by insurance status

	Stage IV at presentation (n = 25 587)				Receipt of Neoadjuvant/Adjuvant radiotherapy (n = 13 648)			
	Unadjusted OR [95% CI]	*P*	Adjusted OR [95% CI]	*P*	Unadjusted OR [95% CI]	*P*	Adjusted OR [95% CI]	*P*
Patients < 65 years								
Insurance status								
Commercial	[reference]	—	[reference]	—	[reference]	—	[reference]	—
Medicaid	2.24 [2.05, 2.45]	<.001	1.74 [1.57, 1.93]	<.001	0.84 [0.75, 0.95]	.003	0.87 [0.77, 0.98]	.021
Uninsured	2.00 [1.79, 2.23]	<.001	1.71 [1.51, 1.94]	<.001	0.74 [0.64, 0.85]	<.001	0.73 [0.63, 0.85]	<.001
Patients ≥ 65 years								
	**Stage IV disease at presentation (n = 21 759)**				**Receipt of Neoadjuvant/Adjuvant Radiotherapy (n = 12 350)**			
Insurance status								
Commercial	[reference]	—	[reference]	—	[reference]	—	[reference]	—
Medicare	1.01 [0.91, 1.12]	.87	0.96 [0.86, 1.07]	.48	0.86 [0.76, 0.96]	.007	0.93 [0.83, 1.04]	.222

Abbreviations: OR = Odds Ratio, CI = Confidence Interval

### 3.3 Association between insurance status and receipt of neoadjuvant or adjuvant radiation

In patients <65 years with locally advanced disease, those with Medicaid (OR: 0.87, 95% CI: 0.77‐0.98, *P* = .021) and the uninsured (OR: 0.73, 95% CI: 0.63‐0.85, *P* < .001) were less likely to receive neoadjuvant or adjuvant radiation therapy as compared to those with commercial insurance. However, in patients ≥65 years, there was no significant difference in the likelihood of receipt of neoadjuvant or adjuvant radiation therapy between patients with Medicare and those with commercial insurance (OR: 0.93, 95% CI: 0.83‐1.04, *P* = .222) (Table 2, Table S2).

### 3.4 Association between insurance status and overall survival

**Figure 2:**
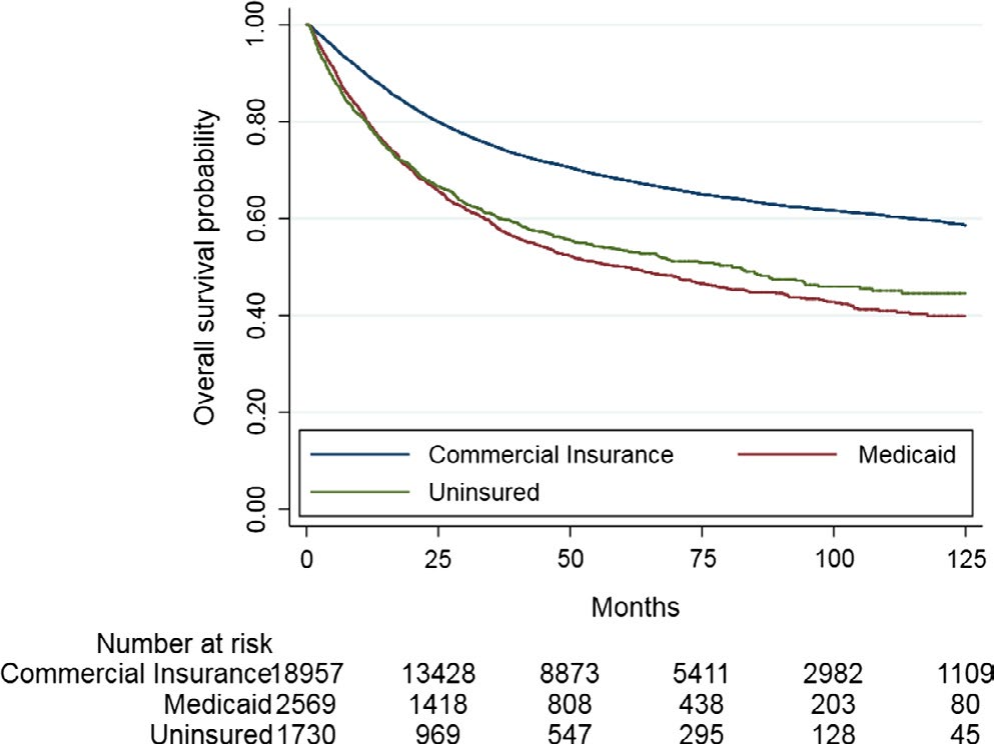
Overall survival as a function of insurance status in patients < 65 years (log rank *P* < .001)

The median follow‐up for the entire cohort was 35 months (IQR: 14‐69 months). In patients <65 years, Medicaid coverage (HR: 1.26, 95% CI: 1.17‐1.34, *P* < .001) and no insurance (HR: 1.30, 95% CI: 1.20‐1.41, *P* < .001) were associated with increased hazards of death relative to commercial insurance (Table 3, Table S3). The estimated 5‐year OS for commercial insurance, Medicaid and no insurance was 59%, 40% and 45%, respectively (log rank *P* < .001) (Figure 2). In contrast, in patients ≥65 years, there was no statistically significant difference in hazards of death between those with Medicare vs. commercial insurance (HR: 1.05, 95% CI: 0.99‐1.11, *P* = .14) (Table 3, Table S3). These findings remained consistent after propensity score matched survival analysis.

**Table 3:** Association of overall survival with insurance status

	Multivariable				Propensity score‐matched cohort	
	Unadjusted HR [95% CI]	*P*	Adjusted HR [95% CI]	*P*	HR [95% CI]	*P*
Insurance status						
Patients < 65 years (n = 23 256)						
Commercial	[reference]	—	[reference]	—	[reference]	—
Medicaid	1.83 [1.72, 1.94]	<.001	1.26 [1.17, 1.34]	<.001	1.19 [1.13, 1.25]	<.001
Uninsured	1.70 [1.57, 1.84]	<.001	1.30 [1.20, 1.41]	<.001	1.23 [1.16, 1.32]	<.001
Patients ≥ 65 years (n = 19 559)						
Commercial	[reference]	—	[reference]	—	[reference]	—
Medicare	1.25 [1.18, 1.33]	<.001	1.05 [0.99, 1.11]	0.14	1.02 [0.97, 1.07]	.13

Abbreviations: HR, Hazards Ratio; CI, Confidence Interval.

## 4 DISCUSSION

In this assessment of a national cancer registry, we demonstrate insurance related disparities in disease presentation, management and outcomes for patients with STS. Specifically, we found that patients <65 years with noncommercial insurance (Medicaid and uninsured) were (a) more likely to present with Stage IV disease at the time of diagnosis, (b) less likely to receive neoadjuvant/adjuvant radiotherapy for locally advanced disease following limb sparing surgery, and (c) had worse survival outcomes compared to patients with commercial/private insurance. In patients ≥65 years, however, there were no significant differences between Medicare and commercial insurance status with regards to these endpoints.

Our findings contribute to the growing body of literature evaluating the impact of insurance coverage on oncological outcomes and quality of care. While previous studies have explored the association between insurance status and outcomes in other malignancies,^9^, ^10^ our study is the first to rigorously examine this question in patients with STS. Furthermore, our study is unique compared to prior studies due its methodology—we stratified patients based on age to specifically assess differences in outcomes between government sponsored insurance plans (Medicaid and Medicare) and commercial insurance, and we examined patterns of receipt of neoadjuvant/adjuvant radiation therapy as a function of insurance status. Our main findings are consistent with previous work that has demonstrated unfavorable patterns of care and inferior outcomes in patients with Medicaid and no insurance.^9^, ^10^, ^11^, ^12^

As with nearly all other cancers, the outcomes of STS patients are highly dependent on the stage of disease at presentation. In general, patients with localized and regional disease can often be treated with curative intent and experience favorable disease outcomes with 5‐year OS ranging from approximately 80%‐90% for patients with stage I disease, approximately 55%‐80% for patients with stage II/III disease and approximately 50% in patients with node positive disease. In contrast, patients with metastatic disease have an estimated 5 year OS <15%.^13^ These results underscore the importance of early detection and a potential lost opportunity for favorable and functional outcomes in patients who present with metastatic disease.

We found that Medicaid and uninsured patients were more likely to present with locally advanced stage (Stage IV) tumors as compared to those with commercial insurance in patients <65 years. This finding is congruent with, and builds upon, previous studies that have demonstrated that patients with nonprivate insurance have an increased likelihood of presenting with advanced breast, prostate, lung, colorectal, head and neck, liver, pancreatic carcinomas, and breast sarcomas as compared to private insurance.^9^, ^12^, ^14^ This observed relationship may plausibly be explained by several factors including lower rates of cancer screening, less frequent interactions with medical system, provider discrimination based on insurance status, receipt of care in underresourced facilities, and cancer serving as a Medicaid‐qualifying event. The latter is substantiated by studies showing that patients who enrolled in Medicaid after their cancer diagnosis present with more advanced disease^14^ and have worse outcomes^15^, ^16^ as compared to those covered by Medicaid prior to their diagnosis.

For patients with locally advanced STS (Stage II/III and non‐metastatic node positive Stage IV), the preferred management strategy is limb sparing surgery with wide resection margins accompanied by neoadjuvant or adjuvant radiotherapy and possibly chemotherapy.^13^ The benefit of adding radiation to limb sparing surgery was demonstrated in two randomized clinical trials, both of which showed a decrease in local recurrence by 20%‐25% with radiation therapy.^17^, ^18^ While these trials were not adequately powered to detect small differences in survival, lower level evidence from a recent Surveillance, Epidemiology and End results database study did demonstrate a modest survival benefit with radiation therapy.^19^ We found that patients <65 years of age without commercial insurance were also less likely to receive neoadjuvant or adjuvant radiation therapy in conjunction with limb sparing surgery for locally advanced disease. These findings are concordant with previous research showing a decreased likelihood of receipt of guideline based care for patients with noncommercial insurance such as Medicaid and the uninsured.^20^

Lastly, we showed that insurance status is an independent predictor of OS in patients <65 years with STS. This finding may be explained by several factors including poor adherence to treatment, lack of social or domestic supports, and decreased access to supportive or ancillary services during treatment, or less follow‐up care in patients with Medicaid or no insurance. Additionally, Medicaid‐managed care plans can limit access to specialized providers and treatment facilities which is particularly important in rare cancers such as STS. Specialized centers have been historically shown to have better outcomes for rare cancers,^21^ including STS.^22^, ^23^ Patients with Medicaid may be less likely to receive high quality care as some specialty providers may be less likely to accept these patients. This unfortunate reality regarding access to specialized care may partly be due to Medicaid reimbursement rates being well below those paid by Medicare and commercial insurance.^24^

A noteworthy finding from our analysis is that Medicare insurance did not adversely impact survival outcomes. In general, patients with Medicare have better access to high quality care compared to Medicaid patients. Consistent with this assertion, studies have shown that patients with Medicare benefit from novel advances in cancer care and their survival outcomes have improved over time, while survival disparities are worsening for Medicaid patients.^25^

Our study has several limitations given its retrospective design and reliance on the content and accuracy of information included in the NCDB. While we attempted to minimize the impact of measured confounders on our outcomes by using propensity score analysis, we were unable to account for several important unmeasured confounders such as adherence to treatment, referral patterns, patient preference, and quality of care received. There may also be issues with misclassification of insurance type. Specifically, the diagnosis of cancer is a qualifying event for Medicaid with the eligibility date assigned as the date of diagnosis. Thus, patients can move from the uninsured to the Medicaid insured group, potentially adversely affecting outcomes for the latter. As the NCDB does not provide information on the duration of insurance, and we are unable to distinguish between those who had Medicaid for many years vs those enrolled at the time of diagnosis. In addition, the NCDB captures a single primary payer, but some patients may have dual insurance coverage or may transition between insurance plans during treatment which would not be captured in the dataset. It is plausible that insurance status is a surrogate for the complex interplay between several socio‐economic and cultural factors that are hard to completely account for in the current analyses. Additionally, our survival analysis is subject to length time and lead time bias as patients with commercial insurance are more likely to have a lower disease stage as well as might have their disease detected earlier even for the same stage than patients with noncommercial insurance. Despite these limitations, the NCDB is a valuable resource for studying rare cancers such as STS, and our overall findings are plausible and concordant with prior work in this field.

In conclusion, we demonstrate rigorously and definitively that insurance status is associated with differences in early diagnosis, receipt of neoadjuvant or adjuvant radiation therapy, and OS for patients with STS in the United States. These insurance related disparities are most prominent in Medicaid and uninsured patients <65 years of age. Further work evaluating insurance coverage quality as well as healthcare policies that lead to such disparities is warranted.

## CONFLICT OF INTEREST

None of the authors have any conflict of interest to disclose.

## AUTHORS’ CONTRIBUTION

Varsha Jain contributed to the conception and design of the article, data analysis and interpretation of the results, drafting the article, critical revision of the article, and final approval of the version to be published. SriramVenigalla contributed to the conception and design of the article, interpretation of the results, critical revision of the article, and final approval of the version to be published. Ronnie Sebro contributed to the interpretation of the results, critical revision of the article, and final approval of the version to be published. Giorgos C. Karakousis contributed to the interpretation of the results, critical revision of the article, and final approval of the version to be published. Robert J. Wilson contributed to the interpretation of the results, critical revision of the article, and final approval of the version to be published. Kristy L. Weber contributed to the interpretation of the results, critical revision of the article, and final approval of the version to be published. Jacob E. Shabason contributed to the conception and design of the article, interpretation of the results, drafting the article, critical revision of the article, and final approval of the version to be published. Jacob E. Shabason is responsible for the overall content as guarantor.

## Data Availability

The primary dataset (National Cancer Database) is available publicly through the American College of Surgeons (https://www.facs.org/quality-programs/cancer/ncdb). The datasets generated and/or analyzed during the current study are available from the corresponding author on reasonable request.
