# Supplementary material for: Association of health insurance status with presentation, treatment and outcomes in soft tissue sarcoma
Source: Cancer Med. 2019 Sep 4;8(14):6295–304. doi: 10.1002/cam4.2441 (PMC6797574; doi:10.1002/cam4.2441)
Supplement: Supplementary file 1 [file CAM4-8-6295-s001.docx]

**Supplementary Tables**

**eTable 1:** Adjusted odds of Stage IV Disease at presentation in patients (a) <65 years and (b) ≥ 65 years; OR = Odds Ratio, CI = Confidence Interval, MPSNT = Malignant peripheral nerve sheath tumor, NOS = Not Otherwise Specified.

| **(a)** |  |  |
| --- | --- | --- |
|  |  |  |
|  | **OR [95% CI]** | **p-value** |
|  |  |  |
| **Insurance** |  |  |
| Commercial | [reference] |  |
| Medicaid | 1.74 [1.57, 1.93] | <0.001 |
| Uninsured | 1.71 [1.51, 1.94] | <0.001 |
|  |  |  |
| **Age (continuous variable)** | 1.00 [0.99, 1.00] | 0.634 |
|  |  |  |
| **Race** |  |  |
| Non-Hispanic White | [reference] |  |
| Non-Hispanic Black | 1.29 [1.16, 1.43] | <0.001 |
| Hispanic | 1.15 [1.01, 1.31] | 0.036 |
| Other | 0.95 [0.80, 1.11] | 0.502 |
|  |  |  |
| **Facility Area** |  |  |
| Metropolitan | [reference] |  |
| Urban | 0.96 [0.86, 1.07] | 0.436 |
| Rural | 1.11 [0.83, 1.47] | 0.477 |
| Unknown | 0.88 [0.68, 1.13] | 0.313 |
|  |  |  |
| **Facility Location** |  |  |
| East | [reference] |  |
| South | 0.97 [0.87, 1.10] | 0.673 |
| Central | 1.02 [0.90, 1.15] | 0.788 |
| West | 0.89 [0.78, 1.02] | 0.097 |
| Unknown | 0.96 [0.80, 1.15] | 0.666 |
|  |  |  |
| **Facility Type** |  |  |
| Non-Academic | [reference] |  |
| Academic | 1.01 [0.92, 1.11] | 0.815 |
|  |  |  |
| **Facility Volume** |  |  |
| 1 | [reference] |  |
| 2 | 1.00 [0.79, 1.25] | 0.983 |
| 3 | 0.82 [0.66, 1.01] | 0.065 |
| 4 | 0.72 [0.59, 0.88] | 0.001 |
|  |  |  |
| **Distance from Treatment Facility (continuous variable)** | 1.00 [0.99, 1.00] | 0.928 |
|  |  |  |
| **Zip Code Education Level** |  |  |
| ≥21% | [reference] |  |
| 13%-20.9% | 0.94 [0.84, 1.05] | 0.264 |
| 7%-12.9% | 0.82 [0.72, 0.93] | 0.002 |
| <7% | 0.84 [0.73, 0.98] | 0.023 |
| Unknown | 1.00 [0.15, 6.52] | 0.997 |
|  |  |  |
| **Zip Code Income Level** |  |  |
| <38,000 | [reference] |  |
| 38,000-47,999 | 1.10 [0.97, 1.23] | 0.126 |
| 48,000-62,999 | 1.09 [0.96, 1.23] | 0.188 |
| ≥63,000 | 0.96 [0.83, 1.11] | 0.600 |
| Unknown | 0.85 | 0.805 |
|  |  |  |
| **Charlson Deyo Score** |  |  |
| 0 | [reference] |  |
| 1 | 1.22 [1.10, 1.36] | <0.001 |
| 2 | 1.42 [1.12, 1.80] | 0.004 |
| 3 | 1.65 [1.09, 2.51] | 0.018 |
|  |  |  |
| **Primary Site** |  |  |
| Head and Neck | [reference] |  |
| Upper Extremity | 0.52 [0.43, 0.63] | <0.001 |
| Lower Extremity | 0.83 [0.70, 0.98] | 0.029 |
| Thorax | 1.41 [1.17, 1.70] | <0.001 |
| Abdomen/Pelvis | 1.59 [1.34, 1.88] | <0.001 |
| Other/NOS | 4.38 [3.57, 5.37] | <0.001 |
|  |  |  |
| **Histology** |  |  |
| Undifferentiated pleomorphic sarcoma | [reference] |  |
| Unclassified | 2.14 [1.82, 2.52] | <0.001 |
| Fibrosarcoma/Myxofibrosarcoma | 0.37 [0.30, 0.46] | <0.001 |
| Liposarcoma | 0.33 [0.27, 0.40] | <0.001 |
| Leiomyosarcoma | 1.39 [1.17, 1.66] | <0.001 |
| Synovial Sarcoma | 2.81 [2.26, 3.50] | <0.001 |
| Angiosarcoma | 1.12 [0.90, 1.38] | <0.001 |
| MPNST | 0.52 [0.45, 0.61] | <0.001 |
|  |  |  |
| **Year** |  |  |
| 2004-2007 | [reference] |  |
| 2008-2011 | 1.47 [1.34, 1.62] | <0.001 |
| 2012-2015 | 1.70 [1.55, 1.86] | <0.001 |
|  |  |  |

*Covariates listed above achieved a threshold significance of p<0.1 on univariate analysis and were included in the multivariable model*

| **(b)** |  |  |
| --- | --- | --- |
|  |  |  |
|  | **OR [95% CI]** | **p-value** |
|  |  |  |
| **Insurance** |  |  |
| Commercial | [reference] |  |
| Medicare | 0.96 [0.86, 1.07] | 0.480 |
|  |  |  |
| **Gender** |  |  |
| Male | [reference] |  |
| Female | 0.84 [0.78, 0.91] | <0.001 |
|  |  |  |
| **Race** |  |  |
| Non-Hispanic White | [reference] |  |
| Non-Hispanic Black | 1.38 [1.20, 1.59] | <0.001 |
| Hispanic | 1.11 [0.91, 1.36] | 0.319 |
| Other | 1.05 [0.86, 1.28] | 0.638 |
|  |  |  |
| **Facility Location** |  |  |
| East | [reference] |  |
| South | 0.82 [0.73, 0.92] | <0.001 |
| Central | 0.95 [0.85, 1.06] | 0.321 |
| West | 0.82 [0.72, 0.93] | 0.002 |
|  |  |  |
| **Facility Type** |  |  |
| Non-Academic | [reference] |  |
| Academic | 0.87 [0.79, 0.95] | 0.003 |
|  |  |  |
| **Facility Volume** |  |  |
| 1 | [reference] |  |
| 2 | 1.08 [0.88, 1.33] | 0.436 |
| 3 | 0.97 [0.80, 1.18] | 0.767 |
| 4 | 0.75 [0.62, 0.91] | 0.003 |
|  |  |  |
| **Distance from Treatment Facility (continuous variable)** | 1.00 [0.99, 1.00] | 0.388 |
|  |  |  |
| **Zip Code Education Level** |  |  |
| ≥21% | [reference] |  |
| 13%-20.9% | 0.90 [0.79, 1.03] | 0.121 |
| 7%-12.9% | 0.82 [0.71, 0.94] | 0.005 |
| <7% | 0.79 [0.67, 0.93] | 0.005 |
|  |  |  |
|  |  |  |
| **Zip Code Income Level** |  |  |
| <38,000 | [reference] |  |
| 38,000-47,999 | 1.04 [0.91, 1.18] | 0.572 |
| 48,000-62,999 | 0.99 [0.87, 1.14] | 0.939 |
| ≥63,000 | 1.00 [0.86, 1.17] | 0.998 |
|  |  |  |
|  |  |  |
| **Charlson Deyo Score** |  |  |
| 0 | [reference] |  |
| 1 | 1.06 [0.97, 1.17] | 0.188 |
| 2 | 1.27 [1.08, 1.51] | 0.004 |
| 3 | 1.21 [0.92, 1.59] | 0.164 |
|  |  |  |
| **Primary Site** |  |  |
| Head and Neck | [reference] |  |
| Upper Extremity | 0.52 [0.42, 0.64] | <0.001 |
| Lower Extremity | 0.89 [0.75, 1.04] | 0.151 |
| Thorax | 1.50 [1.26, 1.79] | <0.001 |
| Abdomen/Pelvis | 2.11 [1.80, 2.48] | <0.001 |
| Other/NOS | 5.67 [4.68, 6.88] | <0.001 |
|  |  |  |
| **Histology** |  |  |
| Undifferentiated pleomorphic sarcoma | [reference] |  |
| Unclassified | 1.98 [1.72, 2.29] | <0.001 |
| Fibrosarcoma/Myxofibrosarcoma | 0.48 [0.38, 0.59] | <0.001 |
| Liposarcoma | 0.36 [0.30, 0.43] | <0.001 |
| Leiomyosarcoma | 1.68 [1.44, 1.97] | <0.001 |
| Synovial Sarcoma | 3.11 [2.27, 4.26] | <0.001 |
| Angiosarcoma | 2.08 [1.72, 2.52] | <0.001 |
| MPNST | 1.64 [1.22, 2.23] | 0.001 |
|  |  |  |
| **Year** |  |  |
| 2004-2007 | [reference] |  |
| 2008-2011 | 1.53 [1.38, 1.69] | <0.001 |
| 2012-2015 | 1.84 [1.67, 2.04] | <0.001 |

*Covariates listed above achieved a threshold significance of p<0.1 on univariate analysis and were included in the multivariable model*

**eTable 2:** Adjusted odds of receipt of neo-adjuvant/adjuvant radiation in patients with locally advanced disease (a)<65 years and (b) ≥ 65 years ; OR = Odds Ratio, CI = Confidence Interval, MPSNT = Malignant peripheral nerve sheath tumor, NOS = Not Otherwise Specified.

| **(a)** |  |  |
| --- | --- | --- |
|  | **OR [95% CI]** | **p-value** |
|  |  |  |
| **Insurance** |  |  |
| Commercial | [reference] |  |
| Medicaid | 0.87 [0.77, 0.98] | 0.021 |
| Uninsured | 0.73 [0.63, 0.85] | <0.001 |
|  |  |  |
| **Gender** |  |  |
| Male | [reference] |  |
| Female | 0.84 [0.78, 0.91] | <0.001 |
|  |  |  |
| **Age (continuous variable)** | 1.00 [1.00, 1.01] | 0.144 |
|  |  |  |
| **Race** |  |  |
| Non-Hispanic White | [reference] |  |
| Non-Hispanic Black | 0.95 [0.84, 1.07] | 0.403 |
| Hispanic | 0.91 [0.79, 1.05] | 0.184 |
| Other | 0.88 [0.75, 1.03] | 0.120 |
|  |  |  |
| **Facility Location** |  |  |
| East | [reference] |  |
| South | 1.03 [0.92, 1.16] | 0.613 |
| Central | 1.26 [1.12, 1.43] | <0.001 |
| West | 1.04 [0.91, 1.19] | 0.526 |
| Unknown | 0.94 [0.78, 1.13] | 0.496 |
|  |  |  |
| **Facility Type** |  |  |
| Non-Academic | [reference] |  |
| Academic | 0.99 [0.89, 1.09] | 0.764 |
|  |  |  |
|  |  |  |
| **Facility Volume** |  |  |
| 1 | [reference] |  |
| 2 | 1.30 [0.98, 1.71] | 0.064 |
| 3 | 1.45 [1.13, 1.87] | 0.004 |
| 4 | 1.31[1.03, 1.67] | 0.030 |
|  |  |  |
| **Distance from Treatment Facility (continuous variable)** | 1.00 [0.99, 1.00] | 0.006 |
|  |  |  |
| **Histology** |  |  |
| Undifferentiated pleomorphic sarcoma | [reference] |  |
| Unclassified | 0.77 [0.67, 0.89] | <0.001 |
| Fibrosarcoma/Myxofibroma | 0.72 [0.61, 0.85] | <0.001 |
| Liposarcoma | 0.80 [0.69, 0.94] | 0.006 |
| Leiomyosarcoma | 0.54 [0.46, 0.63] | <0.001 |
| Synovial Sarcoma | 1.23 [1.01, 1.48] | 0.036 |
| Angiosarcoma | 0.50 [0.38, 0.65] | <0.001 |
| MPNST | 0.89 [0.73, 1.08] | 0.228 |
|  |  |  |
| **Primary Site** |  |  |
| Head and Neck | [reference] |  |
| Upper Extremity | 1.15 [0.95, 1.38] | 0.157 |
| Lower Extremity | 1.28 [1.07, 1.52] | 0.006 |
| Thorax | 0.59 [0.48, 0.71] | <0.001 |
| Abdomen/Pelvis | 0.53 [0.45, 0.64] | <0.001 |
| Other/NOS | 0.58 [0.44, 0.76] | <0.001 |
|  |  |  |
| **Size** |  |  |
| <5cm | [reference] |  |
| 5.1-10 | 1.86 [1.70, 2.03] | <0.001 |
| 10.1-15cm | 1.99 [1.77, 2.23] | <0.001 |
| >15cm | 1.36 [1.20, 1.54] | <0.001 |
| Unknown | 0.24 [0.09, 0.66] | 0.006 |
|  |  |  |
| **Grade** |  |  |
| Grade 1 | [reference] |  |
| Grade 2 | 2.18 [0.73, 6.48] | 0.161 |
| Grade 3 | 3.43 [1.16, 10.19] | 0.026 |
| Unknown/Missing | 2.56 [0.79, 8.28] | 0.115 |
|  |  |  |
| **Receipt of Chemotherapy** |  |  |
| No | [reference] |  |
| Yes | 1.49 [1.36, 1.64] | <0.001 |
| Unknown | 0.74 [0.60, 0.92] | 0.005 |

*Covariates listed above achieved a threshold significance of p<0.1 on univariate analysis and were included in the multivariable model*

| **(b)** |  |  |
| --- | --- | --- |
|  | **OR [95% CI]** | **p-value** |
|  |  |  |
| **Insurance** |  |  |
| Commercial | [reference] |  |
| Medicare | 0.93 [0.83, 1.04] | 0.222 |
|  |  |  |
| **Gender** |  |  |
| Male | [reference] |  |
| Female | 0.81 [0.75, 0.88] | <0.001 |
|  |  |  |
| **Age (continuous variable)** | 0.97 [0.97, 0.98] | <0.001 |
|  |  |  |
| **Facility Location** |  |  |
| East | [reference] |  |
| South | 1.07 [0.96, 1.18] | 0.224 |
| Central | 1.35 [1.21, 1.51] | <0.001 |
| West | 1.16 [1.03, 1.31] | 0.014 |
|  |  |  |
| **Zip Code Education Level** |  |  |
| ≥21% | [reference] |  |
| 13%-20.9% | 1.06 [0.93, 1.21] | 0.402 |
| 7%-12.9% | 1.09 [0.94, 1.25] | 0.252 |
| <7% | 1.09 [0.93, 1.28] | 0.282 |
| Unknown | 0.76 [0.11, 5.05] | 0.777 |
|  |  |  |
| **Zip Code Income Level** |  |  |
| <38,000 | [reference] |  |
| 38,000-47,999 | 0.99 [0.86, 1.13] | 0.842 |
| 48,000-62,999 | 1.05 [0.91, 1.20] | 0.502 |
| ≥63,000 | 1.07 [0.92, 1.25] | 0.376 |
| Unknown | 0.91 [0.14, 5.89] | 0.924 |
|  |  |  |
| **Charlson Deyo Score** |  |  |
| 0 | [reference] |  |
| 1 | 0.86 [0.79, 0.95] | 0.002 |
| 2 | 0.77 [0.64, 0.92] | 0.004 |
| 3 | 0.82 [0.60, 1.12] | 0.206 |
|  |  |  |
| **Histology** |  |  |
| Undifferentiated pleomorphic sarcoma | [reference] |  |
| Unclassified | 0.89 [0.80, 1.00] | 0.051 |
| Fibrosarcoma/Myxofibroma | 0.96 [0.84, 1.10] | 0.580 |
| Liposarcoma | 0.91 [0.79, 1.05] | 0.204 |
| Leiomyosarcoma | 0.59 [0.51, 0.67] | <0.001 |
| Synovial Sarcoma | 0.96 [0.65, 1.40] | 0.816 |
| Angiosarcoma | 0.51 [0.41, 0.62] | <0.001 |
| MPNST | 1.04 [0.77, 1.41] | 0.802 |
|  |  |  |
| **Primary Site** |  |  |
| Head and Neck | [reference] |  |
| Upper Extremity | 1.67 [1.41, 1.97] | <0.001 |
| Lower Extremity | 1.46 [1.33, 1.81] | <0.001 |
| Thorax | 0.90 [0.75, 1.08] | 0.259 |
| Abdomen/Pelvis | 0.64 [0.54, 0.76] | <0.001 |
| Other/NOS | 1.02 [0.78, 1.33] | 0.912 |
|  |  |  |
| **Size** |  |  |
| <5cm | [reference] |  |
| 5.1-10 | 1.74 [1.59, 1.90] | <0.001 |
| 10.1-15cm | 1.67 [1.49, 1.88] | <0.001 |
| >15cm | 1.49 [1.31,1.69] | <0.001 |
| Unknown | 1.91 [0.88, 4.16] | 0.102 |
|  |  |  |
| **Grade** |  |  |
| Grade 1 | [reference] |  |
| Grade 2 | 1.03 [0.33, 3.21] | 0.963 |
| Grade 3 | 1.46 [0.47, 4.54] | 0.517 |
| Unknown/Missing | 1.86 [0.52, 6.72] | 0.343 |
|  |  |  |
| **Receipt of Chemotherapy** |  |  |
| No | [reference] |  |
| Yes | 1.10 [0.95, 1.26] | 0.197 |
| Unknown | 0.64 [0.51, 0.80] | <0.001 |

*Covariates listed above achieved a threshold significance of p<0.1 on univariate analysis and were included in the multivariable model*

**eTable 3**: Factors associated with overall survival in patients (a) <65 years and (b) ≥ 65 years; HR = Hazards Ratio , CI = Confidence Interval, MPSNT = Malignant peripheral nerve sheath tumor, NOS = Not Otherwise Specified.

| **(a)** |  |  |
| --- | --- | --- |
|  | **HR [95% CI]** | **p-value** |
|  |  |  |
| **Insurance** |  |  |
| Commercial | [reference] |  |
| Medicaid | 1.26 [1.17, 1.34] | <0.001 |
| Uninsured | 1.30 [1.20, 1.41] | <0.001 |
|  |  |  |
| **Gender** |  |  |
| Male | [reference] |  |
| Female | 0.88 [0.84, 0.92] | <0.001 |
|  |  |  |
| **Age (continuous variable)** | 1.01 [1.01, 1.01] | <0.001 |
|  |  |  |
| **Race** |  |  |
| Non-Hispanic White | [reference] |  |
| Non-Hispanic Black | 1.03 [0.96, 1.10] | 0.436 |
| Hispanic | 0.78 [0.71, 0.86] | <0.001 |
| Other | 0.90 [0.81, 1.00] | 0.057 |
|  |  |  |
| **Facility Area** |  |  |
| Metropolitain | [reference] |  |
| Urban | 1.06 [0.99, 1.13] | 0.111 |
| Rural | 1.13 [0.95, 1.35] | 0.157 |
| Unknown | 1.18 [1.02, 1.36] | 0.025 |
|  |  |  |
| **Facility Location** |  |  |
| East | [reference] |  |
| South | 0.91 [0.85, 0.99] | 0.019 |
| Central | 1.01 [0.93, 1.09] | 0.831 |
| West | 0.97 [0.89, 1.06] | 0.496 |
| Unknown | 0.91 [0.81, 1.02] | 0.093 |
|  |  |  |
| **Facility Type** |  |  |
| Non-Academic | [reference] |  |
| Academic | 0.94 [0.89, 0.99] | 0.033 |
|  |  |  |
|  |  |  |
| **Facility Volume** |  |  |
| 1 | [reference] |  |
| 2 | 1.09 [0.93, 1.27] | 0.287 |
| 3 | 1.11 [0.96, 1.28] | 0.167 |
| 4 | 1.01 [0.88, 1.17] | 0.835 |
|  |  |  |
| **Zip Code Education Level** |  |  |
| ≥21% | [reference] |  |
| 13%-20.9% | 1.08 [1.00, 1.16] | 0.045 |
| 7%-12.9% | 1.01 [0.93, 1.09] | 0.796 |
| <7% | 0.98 [0.90, 1.08] | 0.752 |
| Unknown | 1.62 [0.84, 3.11] | 0.148 |
|  |  |  |
| **Zip Code Income Level** |  |  |
| <38,000 | [reference] |  |
| 38,000-47,999 | 1.04 [0.96, 1.12] | 0.339 |
| 48,000-62,999 | 0.97 [0.89, 1.05] | 0.401 |
| ≥63,000 | 0.93 [0.85, 1.02] | 0.119 |
| Unknown | 1.14 [0.61, 2.13] | 0.683 |
|  |  |  |
| **Charlson Deyo Score** |  |  |
| 0 | [reference] |  |
| 1 | 1.22 [1.14, 1.31] | <0.001 |
| 2 | 1.56 [1.36, 1.80] | <0.001 |
| 3 | 2.47 [1.94, 3.14] | <0.001 |
|  |  |  |
| **Primary Site** |  |  |
| Head and Neck | [reference] |  |
| Upper Extremity | 0.60 [0.53, 0.68] | <0.001 |
| Lower Extremity | 0.59 [0.52, 0.65] | <0.001 |
| Thorax | 1.00 [0.88, 1.12] | 0.940 |
| Abdomen/Pelvis | 0.85 [0.76, 0.95] | 0.004 |
| Other/NOS | 0.84 [0.74, 0.96] | 0.013 |
|  |  |  |
| **Histology** |  |  |
| Undifferentiated pleomorphic sarcoma | [reference] |  |
| Unclassified | 1.29 [1.17, 1.41] | <0.001 |
| Fibrosarcoma/Myxofibroma | 0.77 [0.67, 0.87] | <0.001 |
| Liposarcoma | 0.78 [0.70, 0.87] | <0.001 |
| Leiomyosarcoma | 0.98 [0.88, 1.08] | 0.681 |
| Synovial Sarcoma | 1.18 [1.05, 1.33] | 0.005 |
| Angiosarcoma | 1.68 [1.47, 1.92] | <0.001 |
| MPNST | 1.72 [1.54, 1.94] | <0.001 |
|  |  |  |
| **Grade** |  |  |
| Grade 1 | [reference] |  |
| Grade 2 | 2.18 [1.94, 2.44] | <0.001 |
| Grade 3 | 4.05 [3.66, 4.48] | <0.001 |
| Unknown/Missing | 3.26 [2.89, 3.68] | <0.001 |
|  |  |  |
| **Size** |  |  |
| <5cm | [reference] |  |
| 5.1-10 | 1.62 [1.51, 1.74] | <0.001 |
| 10.1-15cm | 2.17 [2.00, 2.34] | <0.001 |
| >15cm | 2.58 [2.39, 2.80] | <0.001 |
| Unknown | 1.96 [1.78, 2.17] | <0.001 |
|  |  |  |
| **Presence of Nodes** |  |  |
| Negative | [reference] |  |
| Positive | 1.35 [1.25, 1.46] | <0.001 |
| Unknown/Missing | 0.96 [0.68, 1.36] | 0.815 |
|  |  |  |
| **Presence of Distant Mets** |  |  |
| Negative | [reference] |  |
| Positive | 2.83 [2.64, 3.03] | <0.001 |
| Unknown/Missing | 0.93 [0.53, 1.61] | 0.794 |
|  |  |  |
| **Surgical Intervention** |  |  |
| None | [reference] |  |
| Resection or LSS | 0.47 [0.44, 0.50] | <0.001 |
| Amputation of Limb | 0.70 [0.63, 0.78] | <0.001 |
|  |  |  |
| **Receipt of RT** |  |  |
| No | [reference] |  |
| Yes | 0.83 [0.78, 0.88] | <0.001 |
|  |  |  |
| **Receipt of Chemotherapy** |  |  |
| No | [reference] |  |
| Yes | 0.98 [0.93, 1.03] | 0.474 |
| Unknown | 0.87 [0.75, 1.00] | 0.048 |
|  |  |  |
| **Year of Diagnosis** |  |  |
| 2004-2007 | [reference] |  |
| 2008-2011 | 0.90 [0.85, 0.95] | <0.001 |
| 2012-2015 | 0.87 [0.82, 0.93] | <0.001 |

*Covariates listed above achieved a threshold significance of p<0.1 on univariate analysis and were included in the multivariable model*

| **(b)** |  |  |
| --- | --- | --- |
|  | **HR [95% CI]** | **p-value** |
|  |  |  |
| **Insurance** |  |  |
| Commercial | [reference] |  |
| Medicare | 1.05 [0.99, 1.11] | 0.139 |
|  |  |  |
| **Gender** |  |  |
| Male | [reference] |  |
| Female | 0.90 [0.87, 0.94] | <0.001 |
|  |  |  |
| **Age** | 1.04 [1.04, 1.05] | <0.001 |
|  |  |  |
| **Race** |  |  |
| Non-Hispanic White | [reference] |  |
| Non-Hispanic Black | 0.97 [0.90, 1.04] | 0.405 |
| Hispanic | 0.91 [0.82, 1.01] | 0.087 |
| Other | 0.89 [0.80, 0.99] | 0.031 |
|  |  |  |
| **Facility Area** |  |  |
| Metropolitain | [reference] |  |
| Urban | 0.98 [0.92, 1.04] | 0.434 |
| Rural | 1.02 [0.88, 1.18] | 0.774 |
| Unknown | 0.86 [0.76, 0.98] | 0.027 |
|  |  |  |
| **Facility Location** |  |  |
| East | [reference] |  |
| South | 1.00 [0.95, 1.06] | 0.944 |
| Central | 1.06 [1.00, 1.12] | 0.042 |
| West | 1.02 [0.96, 1.09] | 0.517 |
|  |  |  |
| **Facility Type** |  |  |
| Non-Academic | [reference] |  |
| Academic | 1.00 [0.95, 1.04] | 0.893 |
|  |  |  |
| **Facility Volume** |  |  |
| 1 | [reference] |  |
| 2 | 1.05 [0.94, 1.17] | 0.353 |
| 3 | 0.99 0.89, 1.09] | 0.819 |
| 4 | 0.89 [0.80, 0.98] | 0.016 |
|  |  |  |
| **Distance from Treatment Facility (continuous variable)** | 1.00 [0.99, 1.00] | 0.261 |
|  |  |  |
| **Zip Code Education Level** |  |  |
| ≥21% | [reference] |  |
| 13%-20.9% | 0.97 [0.91, 1.03] | 0.335 |
| 7%-12.9% | 0.97 [0.91, 1.05] | 0.461 |
| <7% | 0.87 [0.80, 0.94] | 0.001 |
| Unknown | 0.57 [0.14, 2.30] | 0.432 |
|  |  |  |
| **Zip Code Income Level** |  |  |
| <38,000 | [reference] |  |
| 38,000-47,999 | 0.92 [0.86, 0.98] | 0.008 |
| 48,000-62,999 | 0.90 [0.84, 0.96] | 0.003 |
| ≥63,000 | 0.87 [0.80, 0.94] | <0.001 |
| Unknown | 1.86 [0.60, 5.78] | 0.285 |
|  |  |  |
| **Charlson Deyo Score** |  |  |
| 0 | [reference] |  |
| 1 | 1.22 [1.17, 1.28] | <0.001 |
| 2 | 1.64 [1.51, 1.78] | <0.001 |
| 3 | 1.91 [1.66, 2.20] | <0.001 |
|  |  |  |
| **Primary Site** |  |  |
| Head and Neck | [reference] |  |
| Upper Extremity | 0.72 [0.66, 0.79] | <0.001 |
| Lower Extremity | 0.68 [0.62, 0.73] | <0.001 |
| Thorax | 0.92 [0.84, 1.00] | 0.058 |
| Abdomen/Pelvis | 0.94 [0.86, 1.02] | 0.121 |
| Other/NOS | 0.76 [0.68, 0.85] | <0.001 |
|  |  |  |
| **Histology** |  |  |
| Undifferentiated pleomorphic sarcoma | [reference] |  |
| Unclassified | 1.15 [1.08, 1.22] | <0.001 |
| Fibrosarcoma/Myxofibroma | 0.88 [0.81, 0.96] | 0.002 |
| Liposarcoma | 0.72 [0.66, 0.77] | <0.001 |
| Leiomyosarcoma | 0.93 [0.86, 0.99] | 0.032 |
| Synovial Sarcoma | 1.19 [1.00, 1.42] | 0.054 |
| Angiosarcoma | 1.52 [1.38, 1.66] | <0.001 |
| MPNST | 1.27 [1.09, 1.47] | 0.002 |
|  |  |  |
| **Grade** |  |  |
| Grade 1 | [reference] |  |
| Grade 2 | 1.49 [1.36, 1.63] | <0.001 |
| Grade 3 | 2.25 [2.09, 2.42] | <0.001 |
| Unknown/Missing | 1.72 [1.55, 1.90] | <0.001 |
|  |  |  |
| **Size** |  |  |
| <5cm | [reference] |  |
| 5.1-10 | 1.46 [1.39, 1.55] | <0.001 |
| 10.1-15cm | 1.90 [1.78, 2.02] | <0.001 |
| >15cm | 2.23 [2.09, 2.38] | <0.001 |
| Unknown | 1.81 [1.66, 1.98] | <0.001 |
|  |  |  |
| **Presence of Nodes** |  |  |
| Negative | [reference] |  |
| Positive | 1.46 [1.35, 1.58] | <0.001 |
| Unknown/Missing | 1.27 [0.86, 1.87] | 0.229 |
|  |  |  |
| **Presence of Distant Mets** |  |  |
| Negative | [reference] |  |
| Positive | 2.52 [2.36, 2.68] | <0.001 |
| Unknown/Missing | 1.35 [0.67, 2.72] | 0.399 |
|  |  |  |
| **Surgical Intervention** |  |  |
| None | [reference] |  |
| Resection or LSS | 0.42 [0.39, 0.44] | <0.001 |
| Amputation of Limb | 0.56 [0.50, 0.62] | <0.001 |
|  |  |  |
| **Receipt of RT** |  |  |
| No | [reference] |  |
| Yes | 0.78 [0.74, 0.81] | <0.001 |
|  |  |  |
| **Receipt of Chemotherapy** |  |  |
| No | [reference] |  |
| Yes | 0.83 [0.78, 0.88] | <0.001 |
| Unknown | 0.77 [0.68, 0.88] | <0.001 |

*Covariates listed above achieved a threshold significance of p<0.1 on univariate analysis and were included in the multivariable model*
